# TCRosetta: An Integrated Analysis and Annotation Platform for T-cell Receptor Sequences

**DOI:** 10.1093/gpbjnl/qzae013

**Published:** 2024-02-08

**Authors:** Tao Yue, Si-Yi Chen, Wen-Kang Shen, Zhan-Ye Zhang, Liming Cheng, An-Yuan Guo

**Affiliations:** Center for Artificial Intelligence Biology, Hubei Bioinformatics & Molecular Imaging Key Laboratory, College of Life Science and Technology, Huazhong University of Science and Technology, Wuhan 430074, China; Center for Artificial Intelligence Biology, Hubei Bioinformatics & Molecular Imaging Key Laboratory, College of Life Science and Technology, Huazhong University of Science and Technology, Wuhan 430074, China; Center for Artificial Intelligence Biology, Hubei Bioinformatics & Molecular Imaging Key Laboratory, College of Life Science and Technology, Huazhong University of Science and Technology, Wuhan 430074, China; Center for Artificial Intelligence Biology, Hubei Bioinformatics & Molecular Imaging Key Laboratory, College of Life Science and Technology, Huazhong University of Science and Technology, Wuhan 430074, China; Department of Laboratory Medicine, Tongji Hospital, Tongji Medical College, Huazhong University of Science and Technology, Wuhan 430030, China; Center for Artificial Intelligence Biology, Hubei Bioinformatics & Molecular Imaging Key Laboratory, College of Life Science and Technology, Huazhong University of Science and Technology, Wuhan 430074, China; Department of Thoracic Surgery, West China Biomedical Big Data Center, West China Hospital, Sichuan University, Chengdu 610041, China

**Keywords:** T-cell receptor, TCR repertoire analysis, Batch search, Annotation, Web server

## Abstract

T cells and T-cell receptors (TCRs) are essential components of the adaptive immune system. Characterization of the TCR repertoire offers a promising and highly informative source for understanding the functions of T cells in the immune response and immunotherapy. Although TCR repertoire studies have attracted much attention, there are few online servers available for TCR repertoire analysis, especially for TCR sequence annotation or advanced analyses. Therefore, we developed TCRosetta, a comprehensive online server that integrates analytical methods for TCR repertoire analysis and visualization. TCRosetta combines general feature analysis, large-scale sequence clustering, network construction, peptide–TCR binding prediction, generation probability calculation, and *k*-mer motif analysis for TCR sequences, making TCR data analysis as simple as possible. The TCRosetta server accepts multiple input data formats and can analyze ∼ 20,000 TCR sequences in less than 3 min. TCRosetta is the most comprehensive web server available for TCR repertoire analysis and is freely available at https://guolab.wchscu.cn/TCRosetta/.

## Introduction

T-cell receptors (TCRs) are T-cell surface protein complexes that could recognize antigenic peptides bound to major histocompatibility complex (MHC) molecules. All TCRs constitute the TCR repertoire, reflecting the immune status of an individual, and are associated with human health. Analyzing the general features of the TCR repertoire (*e.g.*, diversity and V/J gene usage) and discovering key TCR complementarity-determining region 3 (CDR3) sequences can contribute to a better understanding of the immune response [1] and support further clinical research [2]. The diversity of the TCR repertoire is considered a potential biomarker for tracking the response to immunotherapy [3]. The features of the TCR beta chain (TRB) can also assist in early-stage cancer diagnosis [4] and treatment selection [5]. The TRB has been used to detect malignant clones in some blood diseases and has demonstrated better sensitivity and accuracy than traditional methods [6]. Additionally, discovering disease-specific TCR sequences could help TCR-engineered T cells specifically recognize tumor antigens [7].

Several tools for analyzing and visualizing general features of the TCR repertoire, such as tcR [8], VDJtools [9], and VisTCR [10], have been developed. There are also various methods for advanced analyses of the TCR repertoire. For example, TCRdist [11], Grouping of Lymphocyte Interactions by Paratope Hotspots (GLIPH) [12], and Geometric Isometry-based TCR AligNment Algorithm (GIANA) [13] focus on finding antigen-specific TCRs based on sequence similarity. Inference and Generation of Repertoires (IGoR) [14] and Optimized Likelihood estimate of immunoGlobulin Amino-acid sequences (OLGA) [15] can compute TCR sequence generation probabilities. However, these tools can be run only locally in different environments and are challenging for users without programming knowledge. Several online platforms for analyzing the TCR repertoire have also been developed [16–18]; however, all of these platforms require registration and can analyze only the repertoire’s general features. Therefore, providing a user-friendly online server for comprehensive TCR repertoire analysis and providing additional valuable information are essential. In this study, we introduce TCRosetta (http://bioinfo.life.hust.edu.cn/TCRosetta/), a powerful and comprehensive server for analyzing and visualizing the TCR repertoire.

## Method

### Data pre-processing

All the input TCR sequences are subjected to quality control to assure their dependability and quality, based on the following rules: (1) identical CDR3 sequences with different V/J genes will be merged, and only the V/J gene will be retained at the highest frequency; (2) different alleles of the same V/J gene will be merged, and only the gene information will be retained; (3) only sequences, including the V gene and the J gene as well as the entire in-frame CDR3 sequence, will be included; (4) the whole CDR3 sequence will begin with cysteine (C), finish with phenylalanine (F) or tryptophan (W), and contain no stop codon according to the ImMunoGeneTics (IMGT) guidelines [19]; and (5) the CDR3 sequences with lengths smaller than 8 amino acids (AA) or larger than 24 AA will be eliminated.

### Measure TCR repertoire diversity by Renyi entropy

The repertoire diversity quantifies the number of different T-cell clones and their frequencies. We employed the widely used Renyi entropy to estimate TCR repertoire diversity [20]. Renyi entropy can graphically reflect the distribution of abundant clones and rare clones within a specific repertoire and quantify repertoire diversity. Here, $P_{i}$ is the frequency of the sequence in the TCR repertoire, N is the number of unique sequences in the TCR repertoire, and b is the base of the logarithm, which dictates the choice of entropy measurement units. The value of α determines the sensitivity of the diversity index to the sequence abundance in the TCR repertoire, as illustrated in Equation 1. When α≥0, all the sequences have equal weights. The measure of entropy becomes a function of the number of unique sequences but is not dependent on their abundance. When α≥1, the Renyi entropy equals the Shannon entropy.

(1) $$\mathrm{Renyi}\;\mathrm{entropy}=\;\frac{1}{1-\alpha \;}\log_{b}\left(\sum_{i=1}^{N}P_{i}^{\alpha }\right)$$

### Calculation of the TCR repertoire clonality

The description of species abundance equivalence may also be used to quantify the dominance of clones in a TCR repertoire; this is known as clonal evenness [21]. The clonality score is the complement of the clonal evenness score, which can be calculated using the 1 − Pielou index. As shown in Equation 2, a clonality score of 0 indicates a maximally diverse population with even frequencies, whereas a value near 1 indicates a repertoire driven by clonal dominance. Here, $P_{i}$ is the frequency of the sequence, N is the number of unique sequences, and b is the base of the logarithm.

(2) $$\mathrm{Pielou}\;\mathrm{index}=\;-\frac{\sum_{i=1}^{N}P_{i}\log_{b}(P_{i})}{\log_{b}\left(N\right)}$$

### Clustering of the CDR3 sequences and construction of the network

The similarity of CDR3 sequences implies structural resemblance for antigen recognition, which may result in shared antigen specificity [22]. Clustering similar CDR3 sequences is therefore essential for identifying cancer-associated receptors. GIANA is the best tool for clustering large-scale TCR repertoires ($>1\times 10^{6}$ sequences) with high clustering accuracy, specificity, and efficiency [13]. GIANA converts the sequence alignment and clustering problem into a classic nearest neighbor search in high-dimensional Euclidean space. We integrated GIANA into the network analysis in TCRosetta to cluster the CDR3 sequences and subsequently constructed a network. The network analysis workflow contains the following steps: (1) use GIANA to cluster similar CDR3 sequences; (2) use MUSCLE [23] to align sequences obtained in the previous step and then convert them to a distance matrix by computing the Hamming distance between these sequences; (3) convert the distance matrix into an adjacency matrix in which two sequences are linked if their Hamming distance is less than 3; (4) construct a network using igraph (https://igraph.org/) based on the distance matrix from the previous step; (5) calculate the betweenness of each network node using betweenness centrality, which is a measure of the centrality in a graph based on the shortest paths and represents the control of the node in graph theory; (6) combine the betweenness and degree as the weight of a node in the network; (7) use the random walk algorithm [24] to discover communities in the network; and (8) make the sequence logo for each network community using Logomaker [25].

### Calculation of the generation probability of CDR3 sequences

V(D)J recombination creates diverse TCR sequences by assembling different germline V, D, and J combinations and by diversifying junctions between these segments through nucleotide deletions and insertions [14]. Several methods for calculating the probability of CDR3 generation via V(D)J recombination have been developed. OLGA [15] is the best approach for computing the generation probability of CDR3 sequences, balancing the accuracy and efficiency through dynamic programming methods. We calculated the generation probability for CDR3 sequences in TCRosetta using OLGA. The V(D)J recombination model was obtained from the default human T-cell beta chain model of OLGA using the “–humanTRB” option. In addition, we constructed a background by computing the generation probability distribution for 5,000,000 TRB CDR3 sequences randomly selected from the collected healthy samples [26]. The Mann–Whitney U test was subsequently used to examine the difference between the generation probability distributions of the input data and the background.

### Embedding analysis based on the 3-mer CDR3 motif

Previous research has indicated that only partial CDR3 sequences, referred to as “motifs”, contact specific peptides, which is part of TCR specificity [27]. We employed the *k*-mer (k=3) abundance distribution to represent the features of the TCR repertoire based on this discovery. The TCR repertoire is represented by a 3-mer CDR3 motif with an abundance distribution over 20^3^ = 8000 dimensions. The high dimension provides more particular information on the TCR repertoire, but it also contributes much noise to the data. We applied Potential of Heat-diffusion for Affinity-based Trajectory Embedding (PHATE) [28] to embed the TCR repertoire by producing low-dimensional embedding feature vectors, which provide an accurate, denoised representation of biological data that is explicit for visualization and highly scalable in terms of memory and runtime. We constructed a PHATE model, which embedded the 3-mer abundance distribution of the TCR repertoire from seven diseases [breast cancer, coronavirus disease 2019 (COVID-19), Crohn’s disease, melanoma, yellow fever vaccine, classical Hodgkin lymphoma, and non-small cell lung cancer (NSCLC)]. The following steps were performed to construct the model: (1) for each sample, split all CDR3 sequences into 3-mer motifs using a sliding window with a step length of 1; (2) count all motifs to form a CDR3 motif count matrix and exclude samples with either a large (top 20%) or a small number (bottom 20%) of motifs; (3) filter out motifs that do not appear in more than 50% of the disease samples, as a disease-specific motif should be present in majority of the samples of this disease; (4) to exclude non-disease-specific motifs for each disease, use the Mann–Whitney U test to calculate the difference between the distribution of motifs in healthy and disease matrices, and remove motifs from the disease matrix if *P* > 0.05; (5) normalize the motif count matrix from seven diseases using Z score; and (6) use PHATE to construct a distribution model.

### Annotation-based disease state statistics

Available CDR3 sequences with clinical information inspire us to investigate the possibility of annotating clinical information for unknown samples, a function that is absent from all existing web servers. The batch search and annotation function of TCRosetta enables users to search for multiple CDR3 sequences in our reference population consisting of 219,817 annotated CDR3 sequences, which were obtained from three databases: Immune Epitope Database (IEDB) [29], VDJdb [30], and McPAS-TCR [31]. We used Elasticsearch (https://www.elastic.co/), a fast and distributed search engine, to perform a fuzzy search of similar CDR3 sequences with a maximum of 1 mismatch in reference. Only a few CDR3 sequences are potential cancer-associated sequences, and these specific sequences are often cloned at high frequency in the TCR repertoire [32]. Only the top 3000 CDR3 sequences, ordered by frequency, were retained in TCRosetta for annotation.

### Prediction of peptide–TCR binding

TCR recognizes antigen-specific peptides that bind to MHC molecules. Predicting the binding scores between TCRs and peptides is pivotal for the development of immunotherapies and virus vaccines. We assembled a peptide pool of 73 peptides by searching for high-quality (confidence score = 3) peptides from the VDJdb. We predicted the binding scores between TCR sequences and peptides in the pool using ERGO-II. ERGO-II employs natural language processing (NLP) in the prediction model and uses the TCR alpha chain (TRA) CDR3 segment, the TRB CDR3 segment, the MHC typing, and the V and J genes as predictors.

## Results

### Overview

**Figure 1:**
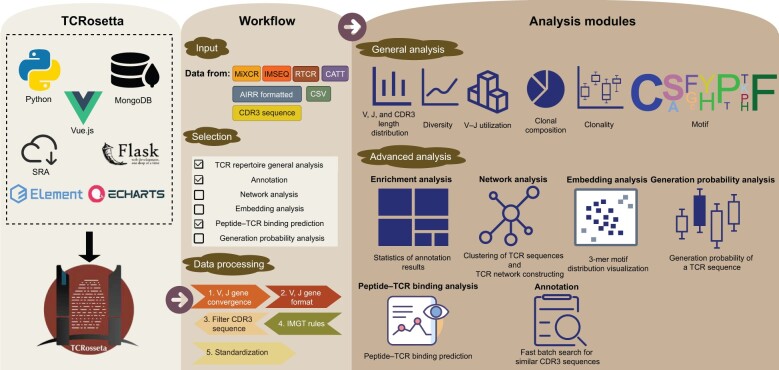
Overview of TCRosetta For the website front end, we used Vue.js and ECharts to build the web server and communicate with user clients. For the back end, we built the web server using Flask and Python 3.7. TCRosetta supports several input formats, and users can select different analysis modules according to their needs. Data processing was used to obtain reliable and high-quality TCR CDR3 sequences. TCRosetta includes general analysis and advanced analysis. TCR, T-cell receptor; CDR3, complementarity-determining region 3.

TCRosetta is a robust online server for analyzing and visualizing the TCR repertoire. Because the majority of TCR sequencing (TCR-seq) data and related studies focus solely on the TRB [33], all TCR repertoire analyses in TCRosetta pertain to the TRB. TCRosetta offers a user-friendly file manager for users to upload and manage data (upload, remove, and clear); it accepts four different types of input data: (1) an output file of V(D)J junction mapping software, such as MiXCR [34], IMSEQ [35], CATT [36], or RTCR [37]; (2) CDR3 sequences; (3) an AIRR-compatible formatted file; and (4) a CSV formatted file. TCRosetta performs analyses depending on user selection to accommodate a variety of user requirements and decrease time expenditure. The input data undergo pre-processing to guarantee data quality. TCRosetta provides general and advanced analyses of the TCR repertoire [38]. The general analyses include repertoire diversity, CDR3 length distribution, V/J gene usage, V–J gene utilization, motif, clonal composition, and clonality. The advanced analyses include network analysis, generation probability analysis, embedding analysis, peptide–TCR binding prediction, and enrichment analysis (**Figure 1**).

### Comparison of TCRosetta with other existing TCR repertoire analysis tools

The rapid expansion of high-throughput sequencing studies on T cells has led to the development of specific tools for TCR repertoire analysis. Downstream analysis of the TCR repertoire is often performed by different tools requiring diverse operating environments and expertise. For example, a suite of tools is often used for TCR repertoire analysis. General analysis may be performed by the tcR package [8], generation probability is calculated by OLGA [15], similar CDR3 is clustered by GIANA [13], and peptide–TCR binding may be predicted by ERGO-II [39]. Although several integrated web servers, such as VDJServer [16], Vidjil [17], VDJviz [18], and IMEX [40], have been developed to solve this problem, the majority of these servers are designed only to analyze the general features of the TCR repertoire. The restrictive format of their built-in TCR repertoire makes it difficult for users to analyze according to their own data format. Importantly, all of these web servers require registration and login (**Table 1**).

**Table 1:** Comparison of TCRosetta with other existing TCR data analysis tools

	Server/software/package	TCRosetta	VDJserver [16]	Vidjil [17]	VDJviz [18]	ImmunoSEQ analyzer	IMEX [40]	Immunarch [8]
Attribute	Type	Server	Server	Server	Server	Software	Software	R package
	Availability	Free	Registration required	Registration required	Registration required	Commercial	Free	Free
	Usage	Online	Online	Online	Online	Local	Local	Local
General analysis	CDR3 length distribution	**√**	**√**	**√**	**√**	**√**	–	**√**
	Diversity	**√**	**√**	–	**√**	**√**	**√**	**√**
	Clonality	**√**	**√**	–	**√**	**√**	**√**	**√**
	V/J gene usage	**√**	**√**	**√**	**√**	**√**	**√**	**√**
	Clonal composition	**√**	**√**	**√**	**√**	**√**	**√**	**√**
	V–J gene combination	**√**	**√**	**√**	**√**	**√**	**√**	**√**
Advanced analysis	Calculate repertoire overlap index	**–**	–	–	–	**√**	–	**√**
	Draw sequence logo	**√**	–	–	–	–	–	–
	Annotate CDR3 sequence	**√**	–	–	–	–	–	**√**
	Cluster and construct network	**√**	–	–	–	**√**	–	–
	Calculate generation probability	**√**	–	–	–	–	–	–
	Track clonotype	–	**√**	**√**	**√**	**√**	**√**	**√**
	Predict peptide–TCR binding	**√**	–	–	–	–	–	–
	Embed sequence motif	**√**	–	–	–	–	–	**√**
Other	Call CDR3 sequence	–	**√**	**√**	–	–	–	–

*Note*: TCR, T-cell receptor; CDR3, complementarity-determining region 3.

TCRosetta is superior to existing web servers, software, and packages for TCR repertoire analysis in the following ways: (1) TCRosetta is the first web server to integrate the majority of known TCR repertoire analysis methods; (2) this approach enables a quick batch search for CDR3 sequences and annotates disease information in reference data; and (3) it offers a user-friendly interface, interactive and downloadable graphs, and personalized analysis options.

### Analysis modules in TCRosetta

The TCRosetta cohort consists of six analysis modules: general, annotation and enrichment, generation probability, embedding, network, and peptide–TCR binding prediction; it is a web server for TCR repertoire analysis that includes many aspects.

#### General module

**Figure 2:**
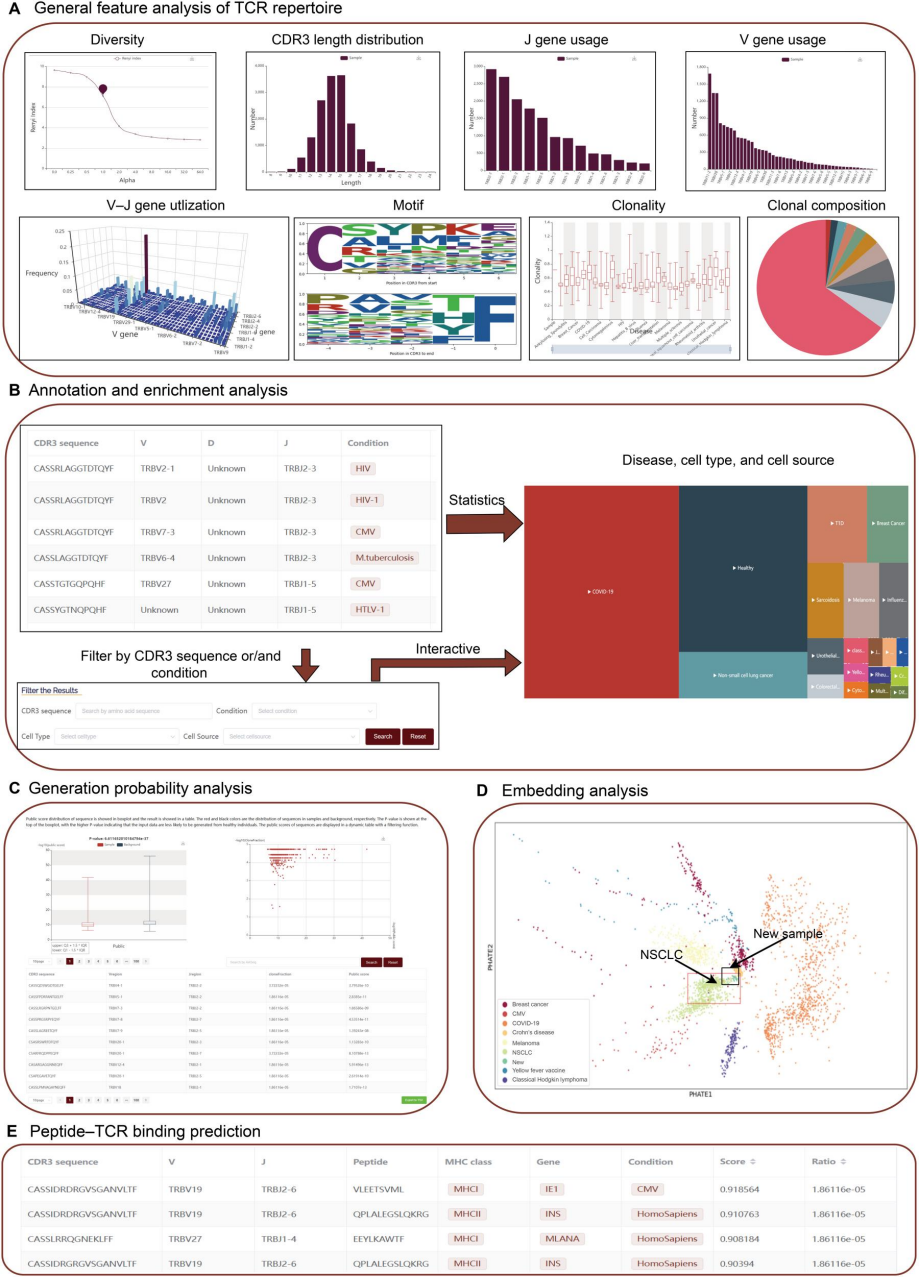
Analysis modules of TCRosetta **A**. General analysis of the TCR repertoire. The TCR repertoire features of the uploaded file include V/J gene usage, diversity, clonality, CDR3 length distribution, motif, and clonal composition. **B**. Annotation and enrichment analysis. The annotation results are displayed in a table. The user can also filter the table by CDR3 sequence, condition, cell type, and cell source. The enriched disease/cell type/cell source distribution of the uploaded data is displayed in an interactive treemap. Different square colors represent different diseases/cell types/cell sources, and the size of the square is the number of unique sequences annotated with this disease/cell type/cell source. Users can further browse the CDR3 sequence and V/J usage by clicking the square. **C**. Generation probability analysis. The generation probability score distribution of the sample is displayed in an interactive box chart. The red and black colors are the distributions of sequences in the repertoire and reference, respectively. *P* value is shown at the top of the boxplot. The relationship between the generation probability score and the frequency of CDR3 sequences in the TCR repertoire is displayed in a scatter plot. The generation probability scores of the sequences are displayed in a dynamic table with a filtering function, and the red points are unique sequences. **D**. Embedding analysis. The result of embedding analysis is on an independent test set. The legend is at the bottom left of the picture, and “New” in the legend represents the samples in the test set. The green points in the red box result from embedding analysis for samples in the test set. Each point represents a sample, and different points represent different diseases. **E**. Peptide–TCR binding prediction analysis. The prediction results are displayed in a dynamic table. MHC, major histocompatibility complex; NSCLC, non-small cell lung cancer.

Some changes in the TCR repertoire, such as diversity and clonality, enable sensitive tracking of dynamic changes in antigen-specific T cells [41]. The general module performs general TCR repertoire feature analysis (**Figure 2**A) including: (1) the CDR3 length distribution; (2) the diversity; (3) the V–J gene utilization; (4) the sequence logo of top 100 frequent CDR3 sequences; (5) the V/J gene usage; (6) the clonality and its distribution; (7) the clonal composition. The general analysis results are displayed in the “General analysis of TCR repertoire” tab of the results page.

#### Annotation and enrichment module

The annotation and enrichment module of unknown CDR3 sequences was used to annotate potential disease states (Figure 2B). This module annotates samples by batch searching similar sequences in disease-related CDR3 sequence references, which were constructed from sequences with known antigen specificities from the VDJdb, IEDB, and McPAS-TCR. Disease distribution in the sample was subsequently calculated based on the search results. The annotation results are displayed in a dynamic table with filtering and exporting functions on the “Annotation by batch search” tab of the results page. The enrichment results are displayed in the “Enrichment analysis” tab of the results page.

#### Generation probability module

Due to the high diversity of the CDR3 sequences, the majority of CDR3 sequences are private to each individual and do not exist in other people. TCRosetta calculates the generation probability of CDR3 sequences and obtains the generation probability distribution using OLGA [15]. The TCRosetta reference, which consists of 5,000,000 TRB CDR3 sequences, was also used to compute the difference in distribution between it and the uploaded data [26]. The generation probability analysis results are displayed in the “Generation probability analysis” tab of the results page (Figure 2C). The generation probability distribution of the uploaded data is displayed in an interactive box chart. The *P* value is shown at the top of the boxplot, with a smaller *P* value indicating a greater difference between the uploaded CDR3 sequence and the reference sequence. The relationship between the generation probability and the frequency of CDR3 sequences is displayed in a scatter chart. The generation probability of sequences is displayed in a dynamic table with a filtering function.

#### Embedding module

CDR3 is the primary region of TCR that contacts peptides, and the distribution of CDR3 sequence motifs may provide disease-specific information [42]. The embedding module splits all the CDR3 AA sequences in the uploaded data into 3-mers to uncover possible disease specificity from the CDR3 motif distribution and obtain a high-dimensional distribution of the motifs. Then, TCRosetta embeds it into a model pre-embedded in PHATE [28]. To evaluate the performance of the embedding analysis, we conducted an independent validation with 38 TCR-seq samples from 16 lung cancer patients. We embedded the dataset in two-dimensional space with our pre-embedded model and found that most of the data points in the dataset were in the region of NSCLC samples (Figure 2D). The embedding analysis results are displayed on the “Embedding analysis” tab of the results page.

#### Peptide–TCR binding prediction module

Many TCRs exhibit a high level of cross-reactivity, with a single TCR binding a large number of peptides and a single MHC peptide binding a large number of TCRs. TCRs that bind the same MHC peptide may share similarities. The prediction module predicts binding scores between TCRs and high-confidence peptides in the VDJdb (Figure 2E). The prediction results are displayed in a dynamic table with filtering and exporting functions on the “Peptide–TCR prediction” tab of the results page.

#### Network module

**Figure 3:**
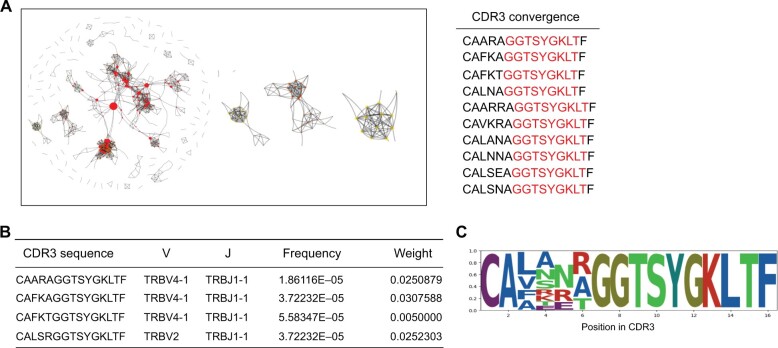
Results of network analysis in TCRosetta **A**. Network of TCR sequences. The size of the nodes represents the weight of the sequence. Different colors represent different communities. The red part of the CDR3 sequence is the conserved residues in this community. **B**. Table of the clustering results. **C**. Sequence logo of CDR3 convergence in the network.

Networks have been used to show immune responses defined by the similarity between sequences [43]. The network module transforms a TCR repertoire into a TCR network by GIANA and igraph, and then divides the network into different communities (**Figure 3**A). Each community consists of similar CDR3 sequences, and the sequence similarity within a community is greater than that outside the community. The clustering results are displayed in a dynamic table with filtering and exporting functions (Figure 3B). To better observe the usage of AA at each position in the sequences in each community, we constructed a sequence logo to show a position weight matrix of the complete CDR3 sequence, which reflects community conservation (Figure 3C). The network, sequence logo, and cluster results are shown in the “Network analysis” tab of the results page.

### Case study: analysis of oral carcinoma by TCRosetta

**Figure 4:**
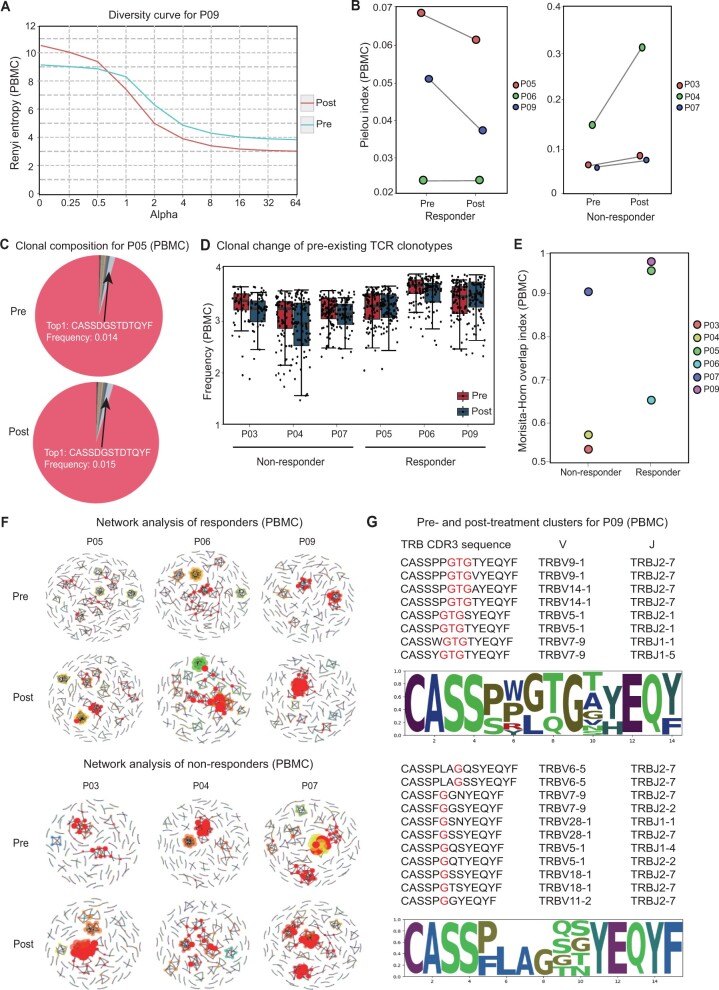
A case study analyzing the TCR repertoire by TCRosetta **A**. The Renyi diversity curve of Patient 9 in the pre-treatment (blue line) and post-treatment (red line) tumor samples. The slope increases as the repertoire’s distribution becomes more monoclonal. **B**. The TCR repertoire clonality of all patient PBMC samples pre-treatment and post-treatment. **C**. The clonal composition of Patient 5 PBMC samples. The top 1 clone and its frequency are shown in the figure. The red part of the sector represents other, which is the total frequency of clones not in the top 10. **D**. Changes in the frequency of pre-existing clones in all patient samples during treatment. **E**. The overlap indices of the TCR repertoire for pre-treatment and post-treatment samples. **F**. The network of all samples. **G**. Pre- and post-treatment clusters and sequence logos for Patient 9 in the PBMC sample. PBMC, peripheral blood mononuclear cell.

Twenty-four subject-matched peripheral blood mononuclear cell (PBMC) and tumor samples from six patients (three responders *versus* three non-responders) from an independent dataset of patients receiving anti-PD-1 immunotherapy for oral carcinoma [44] were used to determine the powerful function of TCRosetta in TCR repertoire analysis. Only the top 20,000 most abundant CDR3 sequences were retained in all the samples to reduce the effect of sequencing depth. All the samples were pre-processed by TCRosetta to ensure data quality and separately uploaded for all the analyses. The results are summarized in **Figure 4**. By comparing the diversity curves of tumor samples from the pre-treatment and post-treatment groups, we found that the diversity of most patients (5 of 6) decreased more quickly after treatment, suggesting that there are more abundant clones in the treated group, such as Patient 9 (Figure 4A). We also detected decreased (2 of 3) or maintained (1 of 3) TCR repertoire clonality in responders and increased (3 of 3) TCR repertoire clonality in non-responders, suggesting that the clonality of the TCR repertoire in PBMCs may be a potential predictor of immunotherapy efficacy (Figure 4B). With respect to the clonal composition of the TCR repertoire, the most abundant clones in some patients expanded during treatment, similar to Patient 5 (Figure 4C). To investigate the changes in pre-existing clones in responders *versus* non-responders before or after immunotherapy, we calculated the frequency of the top 100 pre-existing clones. We found that these clones were not expanded (3 of 3) during treatment in non-responders but were expanded (2 of 3) in responders (Figure 4D), suggesting that pre-existing clones that expand in response to immunotherapy may play a significant role. We also observed greater overlap of productive CDR3 AA sequences in matched PBMC samples (2 of 3) among responders than among non-responders (Figure 4E). According to the generation probability analysis, we observed that the number of responsive PBMCs increased (2 of 3) after treatment, whereas 2 of the 3 non-responsive PBMC samples lost the probability of generation after treatment. By clustering CDR3 sequences in the network analysis module for all patients, we studied the potential cancer-associated TRB CDR3 sequences. We found that the distributions of network nodes in the pre-treatment and post-treatment PBMC samples differed between responders and non-responders (Figure 4F). By analyzing each cluster in the network of Patient 9, we discovered that “GTG” was the conserved motif based on the AA usage frequency in the largest cluster of the pre-treatment sample, which differed from that of the post-treatment sample and may be a favorable motif for antigen binding (Figure 4G).

## Discussion

Analysis of the TCR repertoire helps to elucidate the immune system. However, these bioinformatics analysis tools require extensive skill and have different limitations. Thus, we developed TCRosetta, a comprehensive platform for analyzing the TCR repertoire. Not only could it analyze the features of TCR repertoire and display them in interactive graphs, but it also is the first platform with a batch search and TCR annotation function.

TCRosetta can be applied in many situations. For example, calculating the features (CDR3 sequence length distribution, diversity, V–J utilization, and clonality) of the TCR repertoire can be used as biomarkers for predicting response to immunotherapy. In addition, network analysis could help identify T-cell clones specific to tumors by clustering similar TCR sequences, which could play an important role in some tumor immunotherapies and TCR–T-cell therapies. Moreover, generation probability analysis of TCRosetta may assist in predicting autoimmune disease prognosis by revealing TCR CDR3 sequences shared between individuals associated with self-related immunity.

As a comprehensive interpretation of the TCR repertoire, our study has several limitations. First, TCRosetta mainly studies the features of TRB and ignores TRA. Although TRB plays an important role in tumor antigen recognition, some studies have shown that TRA also has functions in tumor antigen recognition. Second, more than 200,000 reliable TRB CDR3 sequences were integrated with the clinical conditions in the annotation reference, which made it possible to annotate the TCR repertoire. Due to the high diversity of TCRs in the human body (ranging from $10^{12}$ to $10^{18}$), these data may not be sufficient for annotating all TCRs. Third, users cannot upload multiple samples at the same time for comparative analysis.

With the development of TCR studies, we will continue to analyze additional functions of the TCR repertoire and support additional TCR profiling tools. We will further improve the data volume of the reference to ensure that more TCR sequences can be annotated and that users can upload multiple samples for analysis. We believe that TCRosetta will facilitate TCR-related research and clinical applications.

## Code availability

The source code of TCRosetta is available at https://github.com/yttyhhh/TCRosetta_code.

## Data Availability

TCRosetta can be accessed at https://guolab.wchscu.cn/TCRosetta/.

## CRediT author statement

**Yue Tao**: Software, Writing – original draft, Methodology, Resources, Data curation. **Si-Yi Chen:** Software, Methodology. **Wen-Kang Shen**: Data curation. **Zhan-Ye Zhang**: Data curation. **Liming Cheng**: Data curation. **An-Yuan Guo**: Funding acquisition, Writing – review & editing. All the authors have read and approved the final manuscript.
